# Regulatory T Cell Expansion in HTLV-1 and Strongyloidiasis Co-infection Is Associated with Reduced IL-5 Responses to *Strongyloides stercoralis* Antigen

**DOI:** 10.1371/journal.pntd.0000456

**Published:** 2009-06-09

**Authors:** Martin Montes, Cesar Sanchez, Kristien Verdonck, Jordan E. Lake, Elsa Gonzalez, Giovanni Lopez, Angelica Terashima, Thomas Nolan, Dorothy E. Lewis, Eduardo Gotuzzo, A. Clinton White

**Affiliations:** 1 Instituto de Medicina Tropical Alexander von Humboldt, Universidad Peruana Cayetano Heredia, Lima, Peru; 2 Division of Infectious Diseases, Department of Internal Medicine, University of Texas Medical Branch, Galveston, Texas, United States of America; 3 Virology Unit, Department of Microbiology, Institute of Tropical Medicine, Antwerp, Belgium; 4 Department of Medicine, Division of Infectious Diseases, University of California at Los Angeles, Los Angeles, California, United States of America; 5 Department of Pathobiology, University of Pennsylvania School of Veterinary Medicine, Philadelphia, Pennsylvania, United States of America; Swiss Tropical Institute, Switzerland

## Abstract

**Background:**

Human strongyloidiasis varies from a chronic but limited infection in normal hosts to hyperinfection in patients treated with corticosteroids or with HTLV-1 co-infection. Regulatory T cells dampen immune responses to infections. How human strongyloidiasis is controlled and how HTLV-1 infection affects this control are not clear. We hypothesize that HTLV-1 leads to dissemination of *Strongyloides stercoralis* infection by augmenting regulatory T cell numbers, which in turn down regulate the immune response to the parasite.

**Objective:**

To measure peripheral blood T regulatory cells and *Strongyloides stercoralis* larval antigen-specific cytokine responses in strongyloidiasis patients with or without HTLV-1 co-infection.

**Methods:**

Peripheral blood mononuclear cells (PBMCs) were isolated from newly diagnosed strongyloidiasis patients with or without HTLV-1 co-infection. Regulatory T cells were characterized by flow cytometry using intracellular staining for CD4, CD25 and FoxP3. PBMCs were also cultured with and without *Strongyloides* larval antigens. Supernatants were analyzed for IL-5 production.

**Results:**

Patients with HTLV-1 and *Strongyloides* co-infection had higher parasite burdens. Eosinophil counts were decreased in the HTLV-1 and *Strongyloides* co-infected subjects compared to strongyloidiasis-only patients (70.0 vs. 502.5 cells/mm^3^, p = 0.09, Mann-Whitney test). The proportion of regulatory T cells was increased in HTLV-1 positive subjects co-infected with strongyloidiasis compared to patients with only strongyloidiasis or asymptomatic HTLV-1 carriers (median = 17.9% vs. 4.3% vs. 5.9 p<0.05, One-way ANOVA). *Strongyloides* antigen-specific IL-5 responses were reduced in strongyloidiasis/HTLV-1 co-infected patients (5.0 vs. 187.5 pg/ml, p = 0.03, Mann-Whitney test). Reduced IL-5 responses and eosinophil counts were inversely correlated to the number of CD4+CD25+FoxP3+ cells.

**Conclusions:**

Regulatory T cell counts are increased in patients with HTLV-1 and *Strongyloides stercoralis* co-infection and correlate with both low circulating eosinophil counts and reduced antigen-driven IL-5 production. These findings suggest a role for regulatory T cells in susceptibility to *Strongyloides* hyperinfection.

## Author Summary

Human strongyloidiasis varies from a mild, controlled infection to a severe frequently fatal disseminated infection depending on the hosts. Patients infected with the retrovirus HTLV-1 have more frequent and more severe forms of strongyloidiasis. It is not clear how human strongyloidiasis is controlled by the immune system and how HTLV-1 infection affects this control. We hypothesize that HTLV-1 leads to dissemination of *Strongyloides stercoralis* by augmenting regulatory T cell numbers, which in turn down regulate the immune response to the parasite. In our study, patients with HTLV-1 and *Strongyloides* co-infection had higher parasite burdens than patients with only strongyloidiasis. Eosinophils play an essential role in control of strongyloidiasis in animal models, and eosinophil counts were decreased in the HTLV-1 and *Strongyloides stercoralis* co-infected subjects compared to patients with only strongyloidiasis. The proportion of T cells with a regulatory cell phenotype was increased in HTLV-1 positive subjects co-infected with strongyloidiasis compared to patients with only strongyloidiasis. IL-5 is a key host molecule in stimulating eosinophil production and activation, and *Strongyloides stercoralis* antigen-specific IL-5 responses were reduced in strongyloidiasis/HTLV-1 co-infected patients. Reduced IL-5 responses and eosinophil counts were inversely correlated to the number of regulatory T cells. These findings suggest a role for regulatory T cells in susceptibility to *Strongyloides* hyperinfection.

## Introduction

*Strongyloides stercoralis* infects 60 million individuals in tropical and subtropical areas of the world [1]–[3]. It is unique among helminths in that it can complete its life cycle inside a single human host [4]. The clinical presentation of human strongyloidiasis varies with the status of the host's immunity [5]. Immunocompetent individuals develop a chronic, asymptomatic or mildly symptomatic infection. Patients treated with corticosteroids, cancer patients and persons infected with the Human T-cell-lymphotropic virus 1 (HTLV-1), may develop an accelerated form of infection termed hyperinfection, characterized by gastrointestinal and pulmonary hemorrhage, and secondary bacterial infections due to large numbers of parasite larvae migrating from the gut through the lung [6]–[9]. *Strongyloides* hyperinfection has a high fatality rate [10].

Animal models have only provided limited understanding of how the host controls *S. stercoralis*, because the parasite cannot complete the lifecycle in mice [11]. Though murine models are not ideal for this particular parasite, careful studies have suggested a role for innate and adaptative immune mechanisms of control [12]. The innate response requires eosinophils, which kill the larvae [13]. The cytokine IL-5 is essential for development and activation of eosinophils [14]. The adaptive response involves specific antibody production (including IgG and IgE), and granulocytes [15]–[17]. Neutrophils are more important than eosinophils in killing [15]. However, eosinophils are required to generate an optimal antibody response [14], serving as antigen presenting cells [18]. Chemokines attracting eosinophils (eotaxin) and granulocytes (IL-8) are also needed to kill the larvae, presumably by attracting these granulocytes to kill the parasites [19].

There are few studies on the human immune response to *S. stercoralis* infection [7]. Since hyperinfection develops in transplant and corticosteroid-treated patients, some thought that the cellular immune response might play an important response in controlling infection. HIV is a retrovirus that causes depletion of the cellular immune response and acquired immunodeficiency syndrome (AIDS). However, even with severe depletion of CD4 T cells, AIDS patients control dissemination of *S. stercoralis* [20]. Thus, factors other than the cellular immune response are likely more important in control of strongyloidiasis.

HTLV-1 is another human retrovirus endemic in Japan, Africa, the Caribbean and South America [8]. In contrast to patients with AIDS, patients co-infected with *S. stercoralis* and HTLV-1 frequently develop *S. stercoralis* hyperinfection [9],[10],[21]. HTLV-1 and HIV share basic retroviral structures and modes of transmission. Both HTLV-1 and HIV target CD4+ T lymphocytes, but, in contrast to HIV infection where T cells are killed, HTLV-1 activates CD4+ T cells and induces CD4+ T cell proliferation through up-regulation of interleukin-2 (IL-2) and its receptor [8], [22]–[24]. The cytokine profile of T cells isolated from patients with HTLV-1 includes T helper 1 (Th1) cytokines (e.g. IFN-γ) as well as regulatory cytokines (e.g. IL-10 and TGFβ) [25]–[27]. Unlike HIV patients, patients with HTLV-1 are not susceptible to intracellular opportunistic infections [8]. However, disseminated strongyloidiasis is more common in HTLV-1-infected patients than in HIV patients [9],[21].

Regulatory T cells are increasingly recognized as playing a key role in reducing injurious host inflammatory and immune responses [28]. Regulatory T cells control the immune response by different mechanisms: cell-to-cell contact, inhibitory cytokines (e.g. TGF-β, IL-10) and by cytokine deprivation [29]. These cells prevent an excessive immune response and bystander tissue damage during the host response to infections [28]. In murine models of *Leishmania major* infection, regulatory T cells prevent complete elimination of the parasite, yet parasite persistence is required for maintaining the protective immune response [30]. Some studies demonstrate that nematode infections (human and mice) induce regulatory cell expansion [31]. Foxp3 expression levels have been evaluated in patients with HTLV-1 associated myelopathy and asymptomatic carriers with varied results [32],[33]. Foxp3 levels inversely correlate with the rate at which cytotoxic T cells kill HTLV-1 infected lymphocytes in an ex-vivo model [34]. Regulatory T cells have not been studied in HTLV-1 and *S stercoralis* infected patients. We hypothesize that HTLV-I leads to dissemination of *Strongyloides stercoralis* by augmenting regulatory T cells (Treg), which in turn down regulate the immune response against this parasite, allowing for the hyperinfection syndrome.

## Materials and Methods

### Study population and collection of clinical data

The Instituto de Medicina Tropical ‘Alexander von Humboldt’ at the Universidad Peruana Cayetano Heredia (IMT AvH - UPCH) in Lima, Peru is a national referral center for the study of HTLV-1 and its associated diseases. Patients with strongyloidiasis are routinely tested for HTLV-1 infection. Between November 2005 and August 2007, all newly diagnosed subjects with strongyloidiasis were invited to participate. Strongyloidiasis diagnosis was confirmed by stool examination. All those with *S. stercoralis* in stool were tested for HTLV-1 infection by Enzyme-Linked Immuno Sorbent Assay (Ortho HTLVI/HTLVII Ab-capture ELISA, Ortho-Clinical Diagnostics, USA) with confirmatory western blot analysis (INNO-LIA HTLVI/II Score, Innogenetics, Belgium). Consenting patients underwent further clinical and laboratory evaluation, including complete blood count (CBC) with differential, flow cytometry analysis and immunological studies of peripheral blood mononuclear cells (PBMCs). Demographic and symptoms data were obtained from patient interviews and reviews of clinical records. Stool and blood samples were collected at the outpatient clinic at the time of enrollment. In addition, asymptomatic HTLV-1 infected patients and healthy HTLV-1 negative subjects were enrolled as controls.

### Ethics

All participants signed a written informed consent form prior to enrollment in the study. The Institutional Review Board (Comité Institucional de Ética) of the Universidad Peruana Cayetano Heredia in Lima, Perú approved the study protocol and consent forms.

### Stool examination

Routine stool evaluation for ova and parasites included direct examination, and spontaneous and fast sedimentation techniques. Stools were examined for *S. stercoralis* using the Baermann technique modified by Lumbreras [35]. *S. stercoralis* parasite load was reported in a semi-quantitative scale as negative, *1+*, *2+*, *3+* or *4+* according to the number of larvae observed under microscopy by personnel who were blinded to the HTLV-1 status of the participant.

### Flow cytometry

PBMCs were isolated from heparinized blood by density gradient centrifugation (BD Vacutainer CPT Cell Preparation Tube with Sodium Heparin, NJ). Regulatory cells were defined by staining for CD4, CD25, and FoxP3. PBMCs were first stained using Cy-5 conjugated anti-CD4 and phycoerythrine (PE)-conjugated anti-CD25 monoclonal antibodies (BD Biosciences, San Jose, California, USA). After fixing and permeabilizing, the cells were then stained for intracellular FoxP3 using a fluorescein-isothiocyanate (FITC)-conjugated anti-FoxP3 monoclonal antibody (eBiosciences, San Diego, California, USA). Cells were analyzed using a FACScalibur flow cytometer (Beckton Dickinson, Franklin Lakes, New Jersey, USA). Regulatory T cells were identified as CD25+ and FoxP3+ cells among CD4+ cells within the lymphocyte gate. Absolute CD4+ cell counts were performed using a 4 color single platform staining of whole blood cells (anti CD3-FITC, CD4-PE, CD45 PerCP and CD8 APC). Flow cytometry analysis used FlowJo software (V.8.5 Tree Star, Ashland, Oregon, USA).

### Antigen-specific cytokine responses to *Strongyloides stercoralis* antigen

*S. stercoralis* third-stage larvae (L3) were obtained and cleaned as previously described [36]. Briefly, feces from an infected dog was mixed with bone charcoal and cultured at 25°C for 7 days. The L3 were obtained from the fecal-charcoal culture by the use of a Baermann apparatus and allowed to settle in a tube for 30 minutes. The supernatant was then removed and the pellet of L3 was resuspended with an equal volume of 2% liquid low-gelling temperature agar (Sigma Chemical Co., St. Louis, MO, USA) at 37°C. The worm-agar mix was allowed to solidify in the center of a Petri dish and then covered with invertebrate saline. The worms were allowed to migrate out of the solidified agar (leaving most of the bacteria and fecal debris behind) at 37°C for 1 hour. The saline with the clean worms was then centrifuged to pellet the worms. The L3 pellet was frozen at −20°C until needed. Crude antigen was obtained by sonication of L3 larvae and centrifugation, supernatant was collected and protein concentration was determined by colorimetric Bradford assay. PBMCs were cultured in RPMI-1640, 10% Fetal Bovine Serum antibiotic supplemented media (37°C in 5% CO_2_) for 72 hours in the presence or absence of 2-ug/ml crude infective stage *S. stercoralis* larvae (L3) antigen. PBMCs were adjusted to a final concentration of 1×10^6^ cells/ml. Supernatants were collected and stored at −80°C until cytokine analysis.Interleukin 5 (IL-5) was measured by enzyme-linked immuno sorbent assay on the cell culture supernatant following manufacturer's instructions (BD OptEIA, BD Biosciences, San Diego, California, USA).

### Measurement of HTLV-1 proviral load

HTLV-1 proviral load in PBMCs was quantified in the strongyloidiasis and HTLV-1 co-infected patients. HTLV-1 proviral load was quantified using a real-time SYBR Green PCR method, as described previously [37].

### Data analysis

We compared symptoms and parasite load between patients with and without HTLV-1 by Chi square test. Blood results including regulatory T cell numbers and proportions were compared by ANOVA. The IL-5 responses to antigen stimulation were compared by Mann-Whitney U test. The correlation between the IL-5 responses and regulatory T cells were compared by Spearman's rank test.

## Results

A total of 69 subjects were enrolled in the study. Forty-three patients were identified as infected with *S. stercoralis*. Among them, 3 were HIV positive and excluded from further analysis. Of the 40 remaining *S. stercoralis* patients, 27 were infected with *S. stercoralis* alone, and 13 were co-infected with *S. stercoralis* and HTLV-1. Controls included 17 healthy subjects and 9 patients with HTLV-1 with stools negative for *S. stercoralis*.

### Demographics

Demographic data are presented in Table 1. The median age for all patients was 42.0 years (range 22 to 77). Patients with co-infection were slightly older than those with *S. stercoralis* alone (45.0 vs. 39.0 years. p = 0.36). Gender distribution was similar for both groups, with 55% male and 45% female. Patients were originally from Andean, Coastal, and Jungle regions in decreasing order of frequency for both groups. Most patients had visited the jungle within three months of onset of gastrointestinal symptoms.

**Table 1:** Demographics and frequency of clinical signs and symptoms of strongyloidiasis patients with and without HTLV-1 co-infection.

Characteristic	HTLV-1 carriers	Strongyloides+HTLV-1−	Strongyloides+/HTLV-1+	p
	(n = 9)	(n = 27)	(n = 13)	
Gender (M/F)	7/2	15/12	7/6	
Age (years)	40.0	39.0	45.0	
Diarrhea (%)	0/9	16/26 (62)	7/13 (54)	0.65
Constipation (%)	0/9	10/26 (38)	5/13 (38)	1.00
Nausea/Vomiting (%)	0/9	7/26 (27)	8/9 (89)	0.001^*^
Abdominal Pain (%)	0/9	9/26 (35)	6/9 (67)	0.09
Abd. Distension (%)	0/9	10/26 (38)	3/9 (33)	0.78
Flatulence (%)	0/9	11/26 (42)	6/9 (67)	0.21
Weight loss (%)	0/9	10/26 (38)	5/11 (45)	0.69

Clinical data was obtained from newly diagnosed patients with strongyloidiasis by stool modified Baermann sedimentation method and tested for HTLV-1 co-infection. Age and gender were similar among strongyloidiasis patients with or without HTLV-1 co-infection. Gastrointestinal signs and symptoms were more frequently reported among HTLV-1 co-infected patients. Table shows proportion of patients reporting symptoms. Frequencies were compared between *S. stercoralis* groups by Chi square test, * = p<0.05 (Lima, Peru).

### Symptoms

Abdominal pain, diarrhea, flatulence, nausea, vomiting, and increasing abdominal girth were the most common complaints in *S. stercoralis* groups, with over 50% of patients affected. Patients with *S. stercoralis* and HTLV-1 co-infection reported more symptoms than those only infected with *S. stercoralis* (Table 1).

### Stool analysis

**Figure 1:**
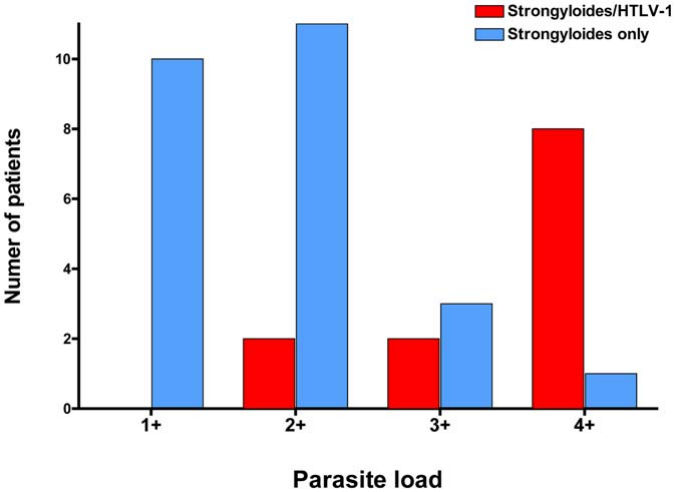
Patients with strongyloidiasis and HTLV-1 co-infection (n = 12) had more *Strongyloides stercoralis* larvae found in stool when compared to strongyloidiasis-only patients (n = 25). A semi-quantitative measure of *Strongyloides stercoralis* parasite load was determined at the time of diagnosis according to the number of larvae observed in stool sediment after Baermann concentration (range from1+ to 4+). Parasite load determination was made before the HTLV-1 status was known. HTLV-1 carriers had higher parasite loads (3+ and 4+) when compared to the non HTLV-1 group (10 out of 12 vs. 4 out of 25 respectively; p<0.001, Chi square test, Lima, Peru).

*S. stercoralis* and HTLV-1 co-infected patients showed more parasites in stool samples compared with patients infected with *S. stercoralis* only. A high parasite burden (3+ or 4+ larvae) was noted in 10 (83%) of twelve of the co-infected patients. Conversely, patients with just *S. stercoralis* had a low burden of disease (84% were 1+ or 2+) (p<0.001, Chi-square test, Figure 1). Other parasites were found in the stool of 51% of all patients, including *Blastocystis hominis* (39%), *Trichuris trichiura* (10%), hookworms (8%), *Giardia lamblia* (5%), and *Ascaris lumbricoides* (2.5%).

### Blood analysis

Median hemoglobin and hematocrit were lower in the *S. stercoralis*/HTLV-1 co-infected group compared to HTLV-1 asymptomatic carriers and *S. stercoralis*-only patients (12.8 versus 13.8 versus 14.1 g/dl, p = 0.11; and 38 versus 41 and 42% p = 0.054 One way ANOVA). Total white blood cell counts and neutrophil counts did not differ significantly among the groups. HTLV-1 asymtpmatic carriers did not show increased eosinophil numbers. Among *S. stercoralis* infected subjects there was a trend towards lower median eosinophils in patients co-infected with HTLV-1 (4% versus 7%; 200 versus 511 cells/mm^3^ (Table 2).

**Table 2:** Blood analysis, flow cytometry and interleukin-5 responses to *Strongyloides stercoralis* larval antigens A peripheral blood sample was obtained at enrollment from all strongyloidiasis patients.

Characteristic	Healthy Controls	HTLV-1 carrier	Strongyloidiasis+HTLV-1−	Strongyloidiasis+HTLV-1+	p
	(n = 17)	(n = 9)	(n = 27)	(n = 13)	
Hemoglobin (g/dl)	-	13.8	14.1	12.80	0.11
Hematocrit (%)	-	41	42	38	0.054
WBC (cells/mm^3^)	-	6200	7500	7000	0.19
Neutrophils (cells/mm^3^)	-	4745	4564	3900	0.69
Eosinophil %	-	1	7	4	0.02*
Eosinophils (cells/mm^3^)	-	47	511	200	0.01*
CD4 count (cells/mm^3^)	721	1166	819	962	0.07
Regulatory T-cell (%)	7.1	5.9%	4.3%	17.9%	<0.0001*
Regulatory T-cell count	53	69	41	233	<0.0001*

Samples were analyzed before HTLV-1 status was known. Numbers represent median of all parameters analyzed. Complete peripheral blood cell count showed lower hemoglobin and hematocrit levels in the HTLV-1 co-infected group. White blood cell, neutrophil, eosinophil, and lymphocyte proportions were not significantly different among groups, although a tendency to lower absolute eosinophil count was observed in the HTLV-1 infected group. Regulatory T cells were significantly increased in patients with strongyloidiasis and HTLV-1 co-infection (* = p<0.05, One way ANOVA, Lima, Peru).

### Flow cytometry analysis

**Figure 2:**
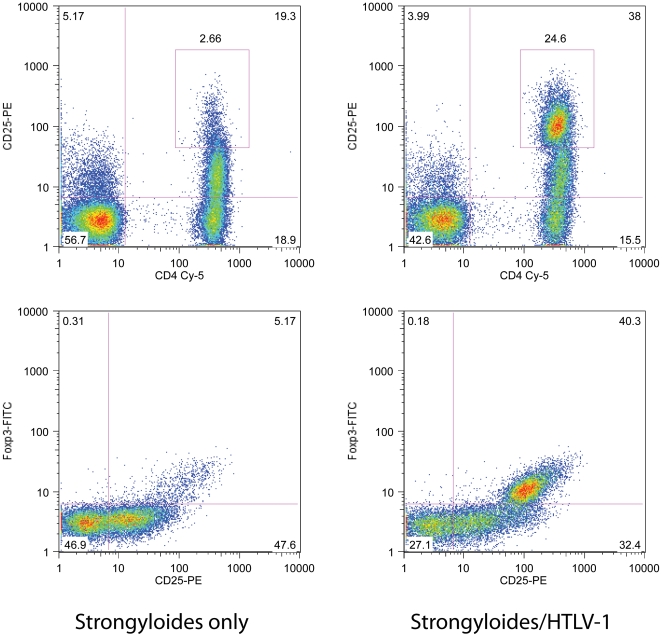
Flow cytometry analysis showing increased proportions of CD4+CD25hi T cells and CD4+CD25+FoxP3+ regulatory T cells in peripheral blood of patients with strongyloidiasis and HTLV-1 co-infection. Representative flow cytometry analysis of peripheral blood mononuclear cells comparing a patient with strongyloidiasis without HTLV-1 co-infection (left) to strongylioidiasis/HTLV-1 co-infected patient (right). Upper figures show CD4 and CD25 staining of cells within the lymphocyte gate. Note population of CD4+CD25hi cells in the HTLV-1 co-infected patient suggesting regulatory T cell phenotype. Lower figures show CD4+ lymphocytes stained with CD25 and intracellular staining with FoxP3 confirming that CD4+CD25hi cells are indeed FoxP3+ (regulatory T cells).

**Figure 3:**
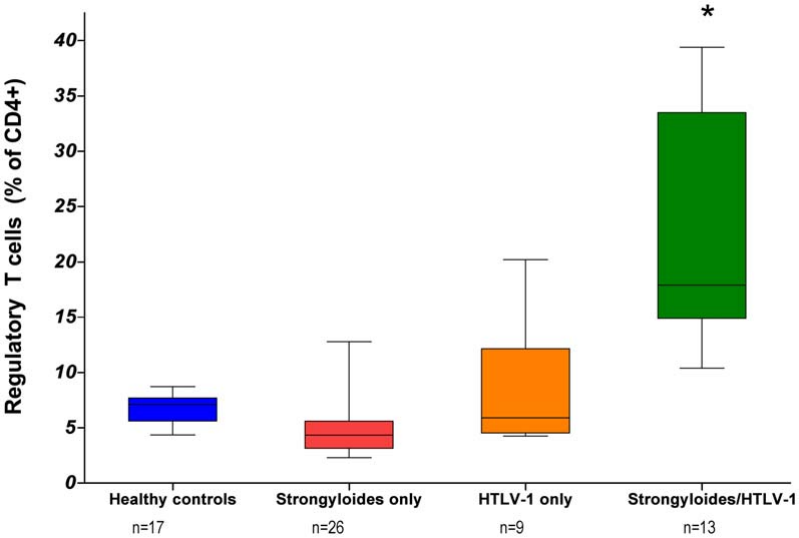
Increased proportions of regulatory T cells in strongyloidiasis/HTLV-1 co-infected patients. Comparison of regulatory T cell proportions (CD25+FoxP3+ among CD4+ T-cells) between healthy controls (blue, n = 17), patients with strongyloidiasis without HTLV-1 infection (red, n = 27), HTLV-1 asymptomatic carriers (orange, n = 9) and strongyloidiasis/HTLV-1 co-infected patients (green, n = 13). Co-infected patients had a significant increase of regulatory T cells compared to non-HTLV-1 patients. (* = P<0.05, One-way ANOVA). Lima, Peru.

The median CD4+ and CD8+ T-cell count were similar in strongyloidiasis patients regardless of their HTLV-1 status (Table 2). By contrast, HTLV-1 co-infected patients had an expanded CD4+CD25^hi^ lymphocyte population. Furthermore, the proportion of CD4+ T-cells with regulatory T-cell phenotype (CD4+CD25+FoxP3+) was significantly increased in the HTLV-1 co-infected patients (median proportion of CD4+ T-cells that were CD25+FoxP3+: 17.9%) when compared to all other groups (p<0.0001 One way ANOVA, Table 2, Figures 2 and 3).

### Interleukin-5 responses to *Strongyloides* antigen

**Figure 4:**
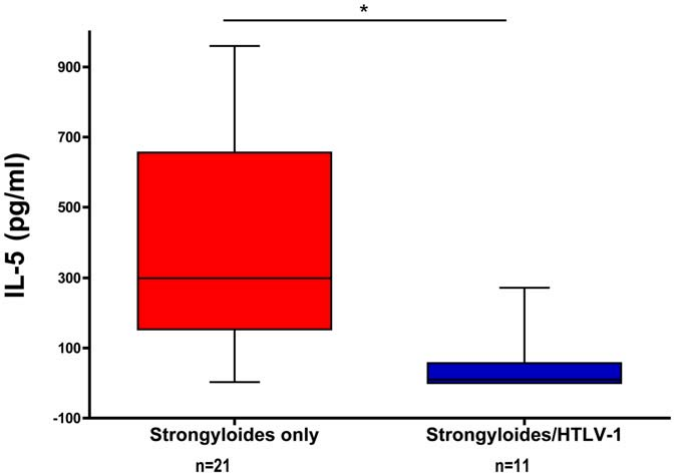
IL-5 responses to larval *Strongyloides stercoralis* antigens are decreased in patients with strongyloidiasis and HTLV-1 co-infection (n = 13) compared to HTLV-1 negative subjects with strongyloidiasis (n = 27). Peripheral blood mononuclear cells from newly diagnosed strongyloidiasis patients were cultured with or without *Strongyloides stercoralis* infective-stage larval antigen. Culture supernatants were analyzed for IL-5 production. Patients with HTLV-1 co-infection had significantly decreased IL-5 responses (* p = 0.0004, Mann Whitney test).

**Figure 5:**
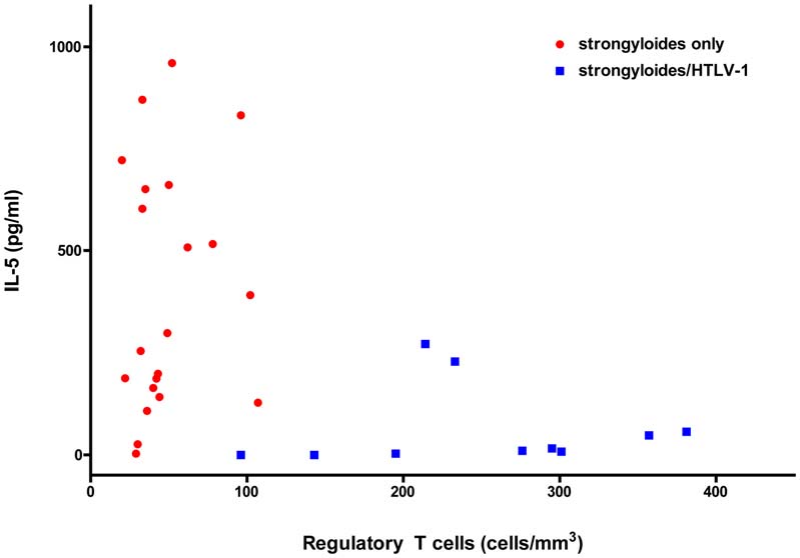
Increased numbers of regulatory T cells correlates with reduced IL-5 responses to *Strongyloides stercoralis* larval antigens. Patients with higher regulatory T cell numbers showed lower IL-5 responses to *Strongyloides stercoralis* infective-stage crude antigen. An inverse correlation was determined by Sperman non-parametric correlation analysis (Spearman r = −0.38, p = 0.03).

PBMC's from strongyloidiasis patients produced IL-5 in response to *S. stercoralis* infective stage larvae crude antigen. However, the IL-5 response was reduced in *S. stercoralis* and HTLV-1 co-infected patients (10 vs 299 pg/ml respectively, p = 0.0004, Mann-Whitney test, Figure 4). An inverse correlation was observed between the number of regulatory T-cells and IL-5 cytokine responses (Spearman r = −0.39, p = 0.03, Figure 5).

### Characteristics of the HTLV-1 proviral load

Proviral load data were available for 9 of the 13 HTLV-1 co-infected patients (mean 1566 copies/10^4^ PBMCs; ranged from 1442 to 5460). These patients had a significantly higher mean proviral load than did asymptomatic carriers (mean 561 copies/10^4^ PBMCs; range 1 to 4773) and were similar to HTLV-1-associated myelopathy patients (mean 1783 copies/10^4^ PBMCs; range 142 to 8641), as reported elsewhere [37].

## Discussion

In this study, we demonstrate that immunocompetent patients with *S. stercoralis* infection have lower worm burdens and higher eosinophil counts compared to patients who are co-infected with HTLV-1 and normal controls. The immunocompetent patients also produce IL-5 in response to stimulation with *S. stercoralis* larval antigens. By contrast, patients with *S. stercoralis* and HTLV-1 co-infection have reduced IL-5 responses to parasite antigens, which may lead to decreased eosinophil count. We also noted increased proportions of CD4+CD25+FoxP3+ regulatory T cells in patients with *S. stercoralis* and HTLV-1 co-infection compared to either normal controls or patients infected with either HTLV-1 or *S. stercoralis* only. The proportion of lymphocytes expressing this phenotype was inversely correlated with antigen-driven IL-5 responses. Thus, augmented regulatory T cell function may explain the defective eosinophil numbers in *S.stercoralis* patients co-infected with HTLV-1.

HTLV-1 is an important risk factor for *Strongyloides* dissemination, but the mechanisms are poorly understood [38]. Previous studies have demonstrated lower eosinophil counts, decreased production of IL-5, and increased production of interferon gamma in patients with *S. stercoralis* and HTLV-1 co-infection [10].

IL-5 is a key growth and activation factor for eosinophils [39]. Eosinophils play a key role in clearance of other helminths larvae [40]. Several mechanisms may be involved including direct cytotoxicity and antibody dependant cellular cytotoxicity [40].

We reasoned that regulatory T cells might be responsible for the reduced IL-5 and eosinophil responses in *S. stercoralis* and HTLV-1 co-infected patients. Indeed, the proportion of CD4+ cells that were CD25+FoxP3+ was dramatically increased in patients with *S. stercoralis* and HTLV-1 co-infection. These levels are higher than have previously been described in humans. Furthermore, those with increased proportions of CD25+FoxP3+ cells had decreased antigen-driven production of IL-5 and lower eosinophil counts. Since HTLV-1 can augment expression of CD25, we also re-analyzed the data without including CD25 expression in the definition of regulatory cells (i.e. CD4+FoxP3+). We also performed an analysis using CD127 expression (low or negative) to define regulatory cells. In all of these analyses, the results were similar. Taken together, these data strongly suggest that Tregs may suppress antigen driven function of T cells, which influences the function of eosinophils.

Pro-viral loads were also elevated in the strongyloidiasis and HTLV-1 co-infected patients, similar to levels increased for other HTLV-1 associated diseases like HTLV-1 associated myelopathy. This suggests that this expanded regulatory T-cell population may be induced by HTLV-1-*S. stercoralis* co-infection or as a response to the proinflammatory effects of these infections. Further studies are needed to determine whether the expansion is in HTLV-1 infection of the Treg cells.

In summary, we have demonstrated that patients with *S. stercoralis* and HTLV-1 co-infection do not control *S. stercoralis* infection as well as patients with *S. stercoralis* only as indicated by the increase in the number of larvae in stool samples. This defect in immune host response is associated with lower eosinophil counts and decreased antigen-driven production of IL-5. This reduced response is inversely correlated with the proportion of CD4 cells, which are CD4+CD25+FoxP3+, suggesting a role for these cells in blunting antigen-driven protective responses. Additional data is needed to determine whether regulatory T cells are increased because they are HTLV-1 infected or as a response to parasite infection, and to explore mechanisms for suppression of eosinophil counts and function.
